# Immunological Markers for PML Prediction in MS Patients Treated with Natalizumab

**DOI:** 10.3389/fimmu.2014.00668

**Published:** 2015-01-05

**Authors:** Caroline Antoniol, Bruno Stankoff

**Affiliations:** ^1^AP-HP, Hôpital Saint-Antoine, Paris, France; ^2^Centre Hospitalier Universitaire de Dijon, Université de Bourgogne, Dijon, France; ^3^Sorbonne Universités, Université Pierre et Marie Curie, UMR S 1127, CNRS UMR 7225, and l’Institut du Cerveau et de la Moelle Épinière (ICM), Paris, France

**Keywords:** JC virus, multiple sclerosis, PML, risk stratification, selectin, effector memory T-cells

## Abstract

Natalizumab (NTZ), a monoclonal antibody recognizing the alpha4 integrin chain, has been approved for the treatment of active multiple sclerosis, but expose to the onset of a rare side effect, progressive multifocal leukoencephalopathy (PML). Estimating the individual risk of PML in NTZ-treated patients is a major challenge, and therapeutic strategies are mainly guided by the overall PML risk assessed by identified risk factors: JC virus (JCV) seropositivity, treatment duration (with peak incidence after 24 months), and the previous use of immunosuppressive therapies. Given that this stratification does not yet allow a precise individual prediction of PML, other predictive markers are needed, and several immunological biomarkers have been described. Quantification of anti-JCV antibody levels may improve individual predictive value, with higher baseline titers indicating increased risk. Other immunological biomarkers such as leukocyte cell membrane markers (CD49d, CD11a, and CD62L), detection of circulating JCV-specific activated T effector memory cells (TEM) or genetic screening have been proposed. In this review, we discuss how recent progress in immunology has paved the way for «new combined monitoring», which will include immunological screening, in NTZ-treated patients.

## Introduction

In 2006, natalizumab (NTZ, Tysabri^®^), a humanized monoclonal antibody targeting the α chain of the α4β1 adhesion molecule, was approved for the treatment of relapsing-remitting multiple sclerosis (RR-MS) (1). This approval was based on pivotal clinical trials showing that NTZ decreased the mean annualized relapse rate by 68% and the risk of disability progression by 42% compared to placebo (1).

This pivotal study not only demonstrated that NTZ therapy could result in a quick benefit for RRMS patients, since the annualized relapse rate was reduced [0.30 (95% CI 0.23–0.40) vs. 0.71 (95% CI 0.55–0.91) *p* < 0.0001] within the first 3 months, but it also emphasized that the efficacy was even more pronounced in patients with highly active disease [0.30 (95% CI 0.17–0.53) vs. 0.94 (95% CI 0.55–1.63)]. Therefore, NTZ emerged as one of the most powerful therapies for active relapsing MS, leading to its rapid and widespread utilization in western countries. However, in contrast with the apparent favorable short-term safety, chronic NTZ use has been associated with progressive multifocal leukoencephalopathy (PML).

First described in 1958 by Astrom and Robertson, PML is a life-threatening opportunistic viral encephalitis induced by JC virus (JCV), and usually occurs in immunocompromised patients (especially HIV+ patients) (2). The overall prevalence of PML among NTZ-treated MS patients was first estimated at 1 case/1000 patients (3–6). However, additional cases have since occurred, and 492 PML cases were reported in 129,100 patients who received at least 1 dose of NTZ up to September 2014. This has resulted in a reviewed estimation of the overall incidence at 3.72/1000 patients (95% CI 3.4–4.06/1000 patients) (Biogen Idec data, September 2014, website http://www.biogenidec-international.com/tysabri.aspx?).

The clinical presentation of NTZ-associated PML is not really distinct from classical PML, and consists in cognitive disorders in more than half of the patients together with motor symptoms, ataxia, neurovisual disturbances, and dysphasia or agnosia in more than 40% of cases (2, 7).

On MRI, lesions are generally large (more than 3 cm) and can affect supratentorial and infratentorial white matter (2). These lesions are usually hyperintense on T2 and FLAIR MRI sequences and hypointense on T1 sequences. Diffusion weighted imaging (DWI) can help in the diagnosis of PML in NTZ-treated patients to differentiate active PML lesions from MS plaques. In contrast with non-NTZ-PML lesions, NTZ-associated PML lesions frequently show gadolinium enhancement (43% of cases) (2, 8). Since “asymptomatic NTZ-PML cases,” defined as subjects with a possible PML lesion identified on MRI without any clinical PML symptom, have been identified among patients who were monitored by systematic MRIs, it is now recommended to perform a regular MRI monitoring (every 3–6 months) among patients at risk for PML, especially after 2 years of treatment (9, 10). Indeed, MRI is very sensitive for the identification of PML recent lesions that can be detected months before first clinical symptoms (9, 10). Interestingly, contrasting with symptomatic PML where lesions tend to involve multiple lobes, «asymptomatic PML»cases are characterized by a more localized disease with unilobar focal lesion affecting predominantly the juxtacortical white matter and the cortical gray matter of the frontal and parietal lobe (11). «Asymptomatic natalizumab-PML cases»are associated with a better survival and functional outcome, further justifying an early detection thanks to the MRI follow-up (12). Once a possible PML case is suspected, the definite diagnosis of PML requires either the detection of JCV DNA in cerebrospinal fluid (CSF) by polymerase chain reaction (PCR) amplification or a brain biopsy to detect JCV DNA on histological examination. Despite improved sensitivity of the newest quantitative PCR techniques for JCV detection in the CSF (13), the result is frequently negative, thus, justifying repeated CSF analysis and the use of ultra-sensitive methods that enable the detection of a very small number of copies with a detection limit up to 10 copies JCV DNA/ml (2, 7). Recently, the determination of CSF anti-JCV antibody index has been proposed as a new immunological marker for early PML detection, which could be an added tool especially in case of low JCV DNA level frequently associated with a “false-negative” PCR for JCV DNA in the CSF (14). The onset of PML in MS has serious prognostic implications, as it leads to death in about 20% of patients or to serious disability in 40% of survivors (15).

In this context of a rare unpredictable and potentially lethal side effect of NTZ, which is usually diagnosed late and for which there is no specific effective curative therapy, great efforts have been directed toward the identification of markers for PML susceptibility among patients treated for MS. The search for markers has mainly relied on the understanding of JCV-induced immunological reactions in infected NTZ recipients, as well as the specific interactions of NTZ with immune cell trafficking.

## Physiopathology of JCV Infection and Reactivation toward PML

JC virus infects glial cells, in particular, oligodendrocytes, lytically (2). JCV consists of a closed, circular, double stranded DNA of 5130 nucleotides with three distinct domains: the early and late coding regions, both controlled by a non-coding control region (NCCR) (2). The early region is responsible for the expression of a series of proteins, called small and large T antigens, which possess regulatory functions in the viral replication cycle (16). The late region encodes three classes of structural proteins VP1, VP2, and VP3, which form the capsid of the virus, and agnoprotein (protein necessary for DNA repair and cell cycle progression) (16).

How this virus infects cells remains partly unknown but it has been suggested that it could use the capsid protein VP1 to enter susceptible cells through binding to *N*-glycoproteins with α2,6-linked sialic acid residues, and to 5-hydroxytryptamine (5-HT) 2A receptors (2). Following cell entry, JCV reach the nucleus via binding to nuclear pore complexes (2).

Primary infection by JCV typically occurs in childhood and is thought to occur in tonsillar tissue after inhalation. JCV-infected tonsillar lymphocytes subsequently carry virions to the kidney and bone marrow considered as the primary sites of viral latency (2). Primary infection by JCV induces both a humoral and a cellular adaptive immune response in the host. While the appearance and maintenance of specific antibodies against the virus, mainly directed against the capsid antigens, will subsequently attest to the primo-infection, they will not confer protective immunity against further JCV reactivation or new infection. In contrasts with the cellular immune response, which is thought to protect the host from JCV reactivation and dissemination, and therefore, prevents PML among immunocompetent subjects. Whereas the immune mechanisms involved in JCV quiescence among immunocompetent hosts are not fully understood, it has been proposed that the protective cellular response mainly relies on the generation of JCV-specific CD8 memory cells, including long-lived effector memory CD8 cells that will ultimately reside within the target organs. However, it has been also shown that CD4 helper T-cells were essential for the optimal generation and maintenance of the pool of memory CD8 T-cells and for optimal antigen reactivation (17, 18). Therefore, in the absence of functional CD4 cells, unhelped CD8 JCV-specific effector cells could fail to prevent JCV replication and dissemination, resulting in the possibility of PML, as often occurs in AIDS (19).

JC virus has the potential to reside and replicate in the kidney, resulting in transient excretion of JCV in the urine in more than one-third of infected subjects. Whether this kidney-specific immune tolerance involves specific Treg populations is still a matter of investigation.

Besides, the kidney, there are still debate concerning the potential role of CD34+ hematopoietic progenitor cells as a reservoir and a carrier for JCV (20). Some authors also reported that JCV could latently infect oligodendrocytes and astrocytes of healthy individuals who do not have PML, suggesting that the brain could also be another site of latency (21, 22).

It is important to note that the latent JCV detected in the urine, named the archetype virus, is always genetically distinct from the JCV detected in the brain and CSF of PML patients, which are neurovirulent JCV mutants (23).

The rearrangement and/or formation of tandem repeats in the NCCR of the JCV genome are certainly required for the pathological infection of glial cells (24). Indeed, the mutations detected in the brain or CSF of PML patients are always genetic rearrangements, such as deletions or duplications, within the NCCR that occur in 100% of cases (23, 25, 26). Mutations in the VP1 structural protein have also been identified in 81% of PML cases (25). Even in PML subjects, the urinary virus usually remains archetypal, and mutations in the NCCR have been observed without VP1 mutations but never the opposite, suggesting that a sequential appearance of intrasomatic JCV mutations (25). These mutations are considered as a necessary condition for the neurovirulence of JCV, but the mechanisms involved in this neurovirulence remain hypothetical: they could contribute to the immune tolerance, provide a kinetic advantage for entering into the CNS, or a selective advantage for CNS replication.

## How Natalizumab Could Affect the Immune Surveillance Against JCV

Natalizumab is a recombinant humanized monoclonal IgG4 antibody that selectively binds to the alpha chain of α4β1 and α4β7 integrin, which are expressed at the cell surface of several hematopoietic cells (i.e., lymphocytes, monocytes, and eosinophils). It prevents the adhesion of cells expressing α4β1 or α4β7 integrins, including activated T-lymphocytes, to endothelial cells expressing vascular cell adhesion molecule-1 (VCAM-1). It thus inhibits diapedesis through the blood–brain barrier (BBB). The overall result is not only an organ-specific deficit in immune surveillance, which is the basis of the therapeutic efficacy, but also underlies the susceptibility to PML.

Several lines of evidence have recently suggested that NTZ could exert differential effects on lymphocyte subsets. This heterogeneity might in theory contribute to individual PML susceptibility. In the peripheral blood, the proportion of CD4+ and CD8+ T-lymphocytes under NTZ treatment varies among studies. Some show a stable rate (27, 28) or a non-significant decrease (29), others report an increased percentage of T-cells (1.3 times) (30), but without changing the CD4/CD8 ratio (30) contrasting with the decrease in the CD4/CD8 ratio reported in the CNS (31). In the CSF, CD4+, and CD8+ T-cells, as well as CD19+ B-cell and CD138+ plasma cells were found lower in NTZ-treated patients compared with untreated MS patients or healthy subjects, this lower rate persisting 6 months after NTZ cessation, but returning to normal levels 14 months after NTZ cessation independently of any clinical or radiological rebound phenomenon (29, 32).

A pronounced and long-lasting decrease in CD49d (α4) expression by circulating T-lymphocytes is induced by NTZ, contrasting with a less pronounced decrease in CD29 (β1) (27, 33). When blood and CSF counts have been assessed in parallel, a lack of CD49d expressing cells was observed in the CSF as expected. However, an increased expression by peripheral T-cells of the selectin PSGL-A, which is involved in the rolling of T-cells, was also discovered. This increased expression could facilitate entry into the CNS of lymphocyte subsets that are not exclusively dependent upon the VLA4–VCAM1 interaction via the choroid plexus. This could finally result in the maintained entrance of MCAM-positive lymphocyte subsets such as Th17 cells, which have been identified in the CSF of NTZ-treated patients (33). Whether the differential impact of NTZ on T-cell subset trafficking could be involved in PML susceptibility remains purely speculative.

Despite the profound impact of NTZ on T-cells, its action cannot be simply reduced to inhibition of the passage of activated T-lymphocytes through the BBB. Indeed, it seems to have a broader immunological action on various players of the innate and adaptive immune system such as antigen presenting cells (APC) and natural killer (NK) cells.

In order to be presented to T-cells, the antigen must be picked up by APC such as dendritic cells (DCs), B lymphocytes, and macrophages. NTZ appears to have an effect on two of them, B lymphocytes and DC. The number of CD209+ cells was found to be decreased in the cerebral perivascular spaces of NTZ-treated subjects as was the expression of MHC class II molecules (expressed on the surface of DC) (34). The decreased expression of VLA4 on myeloid DCs (mDCs) and to a lesser extent on peripheral blood plasmacytoid DC (pDC) was reported in patients on NTZ (35). *In vitro*, NTZ decreased the functional capacity of pDCs to stimulate CD4+ T-cells (35), thus, to CD8+ T-cells. These results suggested that *in vivo*, NTZ may modulate the ability of DC to induce a CD4+ T-cell-specific response (35) with a subsequent deficient anti-JCV response.

CNS resident DC are indeed key players in the immune defense against JCV: following priming by CNS replication, tissue imprinted DC migrate toward the draining lymph nodes where they contribute to the activation of T-cell populations, including naive T-cells, central memory cells, and effector T-cells, all of which will recirculate toward the CNS and support the anti-JCV effector function of the long-term CNS resident pool of memory effector cells (36). Along the same lines, interesting results on MHC class II molecules have been recently emphasized. The HLA-DR1*15 haplotype, which is also the most strongly associated genetic risk factor of MS, has been linked with a protective effect against JCV infection, whereas a strong negative association was reported for the HLA DQB1*06:03 haplotype (37, 38). Genetic characterization of the HLA complex could therefore help to determine an individual’s risk of PML.

Natalizumab exerts some effects on B-cells: (i) it mobilizes CD34+ progenitor cells out of bone marrow into peripheral blood and elevates persistently circulating CD19+ B-cells, particularly CD19+ CD10+ pre-B-cells for extended periods (20, 39); (ii) it leads to a global increase in the proportion of CD 20+ B-cells whereas the percentage of B-cells expressing VLA4 is reduced (27); and (iii) it modifies B subpopulations with a decrease in naive B-cells and an increase in memory B-cells (CD19+CD27+IgD−) (30). As memory B-cells need the VLA-4/VCAM-1 interaction to be contained within the spleen, blocking this interaction could lead to the release of memory B-cells from the spleen.

Though still a matter of debate, it has been highlighted that B-cells and CD34+ progenitors could play a role in the pathophysiology of PML, as these cells might be a reservoir of JCV (20, 39–41). The release of pre-B-cells and CD34+ stem cells from the bone marrow to the periphery could be one of the mechanisms behind an increase in JC viremia, thus, promoting the emergence of PML (42). Since 1992, special attention was paid to the capacity of CD34+ to be infected by JCV, allowing the transfer of JCV into lymphocytes (40, 43). This hypothesis has been reinforced by recent *in vivo* findings, emphasizing the detection of a higher JCV DNA load in CD34+ cells and in CD19+ cells among NTZ-treated patients, suggesting that subclinical JCV reactivation may occur in blood. Whether the detection of cellular JCV DNA could be used for risk stratification algorithms remains to be explored (39, 41). Nevertheless, the JCV DNA load detected was very low in these patients and JCV DNA copies have also been identified in healthy subjects and in MS patients during interferon therapy, impacting the specificity of such findings (44).

B-cells may also play a role in the transport of JCV within the CNS. Indeed, the infection of pre-B-cells by the JCV archetype (form of the virus during primary infection) might allow changes in the regulatory region during the development and maturation of B-cells, in particular, via the expression of RAG 1 and RAG 2 recombinases [needed for somatic recombination of V (D) J rearrangement of immunoglobulin] and the AID (activation-induced cytidine deaminase) enzyme, which allows somatic hypermutation, gene conversion, and immunoglobulin class switching (45). Therefore, the genesis of B-cells and antibodies could alter the genotype of JCV and allow it to be expressed within B-cells and glial cells (45). Finally, assuming that DRB1*06:03 seems to have a positive association with anti-JCV antibody status, one can speculate that less effective viral control can lead to a chronic higher stimulation of B-cells and an increase in anti-JCV antibody levels (38).

## JCV-Specific Antibodies and PML Prediction

Estimating the individual risk of PML remains a major challenge, and therapeutic use of NTZ in MS is guided by the overall estimation of the probability of developing PML. Since the approval of NTZ, three main risk factors for PML have been identified and are generally used in a clinical setting: the presence of JCV-specific antibodies, the increasing duration of treatment (with a peak at 24 months), and a history of immunosuppressive therapy. Combining the three risk factors result in a PML probability of 1:91 (46), which largely exceeds the overall probability of 3/1000. However, these risk factors are far from satisfactory to achieve a reliable individual prediction of PML, a goal that remains a crucial challenge for clinicians.

**Figure 1:**
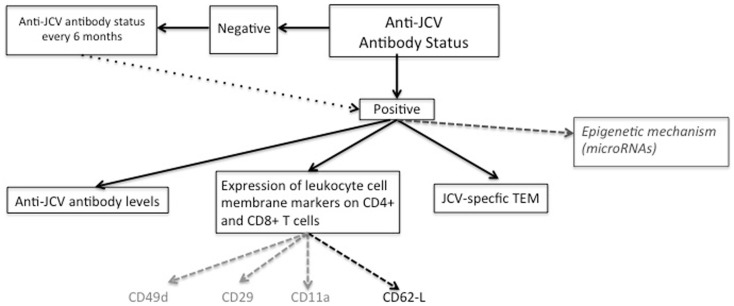
**Immunological markers to estimate individual PML risk in NTZ-treated patients**.

The absolute prerequisite for PML development is previous contact with JCV, which can be confirmed retrospectively by the presence of JCV-specific antibodies (47) (Figure 1). Hence, a two-step serological assay consisting of an enzyme-linked immunosorbent assay (ELISA) was developed a few years ago. This assay is based on the detection of antibodies against JC virus-like particles (VLPs). In 2013, this technique was expanded to a second-generation JCV antibody ELISA, which provides better sensitivity and specificity (48) by pre-coating the JC VLPs on microtiter plates (48). In this second assay, reproducibility has been improved thanks to a ready-to-use kit, and the cross-reactivity of BK virus-specific antibodies appeared to be very low. The proportion of false negatives is low at around 2.5% (49). On the whole, 50–60% of multiple sclerosis patients are positive for JCV antibodies (48, 50, 51) but only a very small proportion will develop PML, illustrating that stratification according to JCV antibodies does not yet allow a precise individual prediction of PML. Interestingly, JCV IgG seroprevalence increases with age (up to 68% of people by the age of 59) (24). Depending on the studies and the serological test used, approximately 50–90% of adults have been exposed to JCV whereas 19–27% of these shed JCV in their urine (2).

Anti-JCV antibody levels have been proposed to improve the prediction of PML in NTZ-treated patients. An increase in serum anti-JCV antibody levels was observed at the onset of PML, and interpreted as being related to a new site of lytic infection, and/or to the pathogenic transformation of the virus (52).

Starting out from this observation, a quantitative anti-JCV antibody index is currently being investigated to determine its putative predictive value, with the hypothesis that increasing titers could reflect an increasing risk (53) (Figure 1). One point worth mentioning is that the anti-JCV antibody index does not correlate with duration of exposure to NTZ (53). A major restriction that emerged from the first studies was that the kinetics of JCV antibodies could not be predictive among patients with prior use of immunosuppressive drugs, who are those with the highest PML risk: indeed, there was no difference between PML and non-PML patients for the distribution of the anti-JCV antibody index in this subpopulation (53).

Among patients without prior immunosuppressant use, a link between a higher anti-JCV antibody index and a greater risk of PML was suggested (53) and a cut-off point of 1.5 for the antibody index was proposed to identify patients with a higher risk. More precisely, among patients without prior immunosuppressant use and with an anti-JCV antibody index at or below thresholds of 0.9–1.5, the risk of PML remained low during the first 2 years of treatment (approximately 0.1/1000 patients, i.e., the same as anti-JCV antibody-negative patients), and extended from 0.3/1000 (if index below 0.9) to 1.3/1000 (if index between 0.0 and 1.5) after 25 months of treatment. Importantly, the cut-off of 1.5 seems to be the value “not to be exceed.” Above that threshold, the risk of developing PML was estimated to be 1/1000 patients during the first 2 years of treatment and ranged from 8.1 to 8.5/1000 after 25 infusions. These recent results could have a significant impact for the management of NTZ-treated patients. Having an anti-JCV antibody index <0.9 corresponds to a very low risk of PML, which could allow considering therapy maintenance despite seropositivity. On the contrary, having an anti-JCV antibody index >1.5 could be interpretated as a «red flag», encouraging reconsidering the risk-benefit balance and to increase clinical and MRI surveillance if therapy is pursued. Finally, an index between 0.9 and 1.5 might be considered as a «gray area»in which PML risk can increase with time (up to 1.3/1000 patients), which requires to discuss continuation of therapy at the individual level. Briefly, the lower anti-JCV antibody index, the higher safety seems to be.

However, to date, this quantitative approach has still to be further validated and is not yet recommended for general use, and clinical decisions are still mainly based on the positive or negative serological status.

Initial interpretations of this serological test suggested that a negative status could be used to reassure patients as the probability of developing PML is very low at <1/10000. A longitudinal serological follow-up from the combined AFFIRM and STRATIFY-1 studies showed, however, that only 87% of patients who were anti-JCV antibody negative at baseline remained consistently negative every 6 months over a period of 18 months (53) meaning that nearly 13% could become positive during follow-up. Interestingly, 39% of them reverted back to negative at least once during the 18 months whereas 69 and 74% remained below the anti-JCV antibody index threshold of 0.9 and 1.5, respectively (53). This was interpreted as relating either to an initial false negative, seroconversion during follow-up or low levels of antibodies, which could fluctuate slightly around the positivity cut-off. Therefore, negative JCV serology at treatment initiation is not sufficient for long-term reassurance and the current recommendation is to test patients every 6 months for antibody levels (54).

## New Approach Based on Leukocyte Cell Membrane Markers

Several immunological biomarkers related to cell surface molecules, mainly those belonging to the integrin and selectin families and involved in cell trafficking through the BBB, have been investigated for their predictive value (Figure 1).

Several modifications in integrin expression have been pointed out following NTZ initiation. Down-regulation of CD49d and CD29 (subunit of VLA-4) expression on both CD4+ and CD8+ T-cells has been shown in patients treated with NTZ, as early as the first months of therapy and lasting several years (55). The decreased expression of CD11a (the α chain of lymphocyte function-associated antigen 1 or LFA-1) on both CD4+ and CD8+ T-cells was detected at 24 months, which corresponds to the peak incidence of PML (55). To a lesser extent, a decrease in CXCR3 was also observed after 1 year. Assuming that VLA-4 and LFA-1 are crucial for the migration of Th1 and Th17 lymphocytes into the central nervous system (56), their decreased function could be involved in the inhibition of anti-JCV-specific T-cell trafficking in the CNS, thus, leading to PML (57). However, these markers have not yet demonstrated their predictive value in a clinical setting.

The role of L-selectin, named CD62L, a cell adhesion molecule expressed on T-lymphocytes, has recently been highlighted. L-selectin plays a crucial role in the initial step of the adhesion cascade, the capture of leukocytes from the blood and the rolling along the vascular endothelium (58). Moreover, getting rid of L-selectin from activated T-cells prevents their reentry into peripheral lymph nodes, which could have an impact on T helpers.

A recent study demonstrated that the proportion of CD4+ T-cells expressing L-selectin was lower in long-term NTZ-treated patients than in untreated MS patients or healthy controls, and an unusually low percentage of CD4+ T-cells expressing CD62L was associated with a higher risk of developing PML (59). In NTZ-treated patients who developed PML, a dramatic decrease in CD62L+ cells has been described prior to PML onset in a small number of cases (59). No correlation seems to be observed between CD62L-expressing-cells and JCV seropositivity or prior use of immunosuppressive drugs (59). One limitation of this study is due to the fact that the PBMC were cryopreserved, which could have severely modified cell expression of selectins. Therefore, a confirmatory study on fresh cells is awaited. To explain the possible link between the decrease in CD62L and PML, several hypotheses have been put forward. As CD62L is involved in T-cell trafficking though the BBB, it could contribute to preventing the entrance of anti-JCV T-cells into the CNS. CD62L is also involved in the final function of T effector cells in the CNS, thus, in mediating demyelinating damage (60). Therefore, the lack of CD62L could have a negative impact on the capacity of JCV-specific T-cells to kill infected oligodendrocytes. Finally, CD62L expression is crucial for lymphocyte migration toward lymph nodes (61), and decreased expression could also impair the activation of naive T-cells by APC in lymph nodes (59). Consequently, NTZ seems to decrease not only T-cell trafficking but also their activation by APC at the same time, thus, leading to impairment of immune defenses against viral infection, especially JCV infection.

While these results are awaiting confirmation in prospective studies, it has been proposed that the bi-yearly monitoring of CD62L expression on D4+ T-cells after 18 months of NTZ treatment could help to evaluate the individual risk of PML (59). Whether the combined monitoring of L-selectin, CD49d, and CD11a could predict the individual risk of PML among NTZ-treated patients is an important question to be answered in future studies.

## Dynamics of Peripheral JCV Effector Memory T-Cells as a Marker of JCV Reactivation

The detection of circulating JCV-specific activated effector memory T-cells (TEM) has been proposed to improve the identification of patients with a high risk of PML (62) (Figure 1). One underlying idea was that following reactivation of the JCV and production of JCV antigens, which could occur in the brain, APC will migrate toward the draining lymph nodes and promote the activation of anti-JC T-lymphocytes, including effector memory T-cells. Once these effector cells are activated they migrate into the peripheral circulation where they will remain, as their entrance in the CNS is inhibited by NTZ. They can therefore be detected thanks to their release of INF-γ, which can be examined by an enzyme-linked immunosorbent spot assay (ELISPOT). In 2009, Chen and colleagues first tried to determine JCV protein VP1-specific T-cell responses thanks to an interferon gamma immunospot assay (63). PBMCs were isolated from patients treated for more than 18 months and were exposed to four pools of overlapping peptides to cover the entire JCV protein VP1. A decrease in spot-forming units (SFU) was observed between 6 and 12 months for three peptide pools, suggesting that NTZ could have a negative effect on the virus-specific T-cell response (63).

Recently, a new test for the detection of JCV-specific effector memory T-cells (TEM) using an ELISPOT procedure has been developed and assessed among NTZ-treated patients (62). A threshold for positivity had been defined from healthy subjects, and the initial assessment of this ELISPOT test on a patient with a biopsy proven PML but negative JCV PCR in the CSF showed a strong positivity for circulating TCM, suggesting that the presence of detectable JCV-specific TEM accompanied the onset of PML. Screening for JCV-specific TEM responses in “non-PML subjects,” belonging to three groups (RR-MS under NTZ, untreated RR-MS, and healthy donors) showed that the frequency of JCV TEM responses increased with the time on NTZ, with a peak at 24 months, meaning that prolonged NTZ treatment is sometimes associated with JCV reactivation. In addition, most of the patients who developed PML showed high titers. TEM positivity was detected almost exclusively in patients with positive serology for JCV, but identified only a minority of seropositive subjects (16.1%) (62). Therefore, the detection of JCV-specific TEM using this ELISPOT procedure could help to identify the minority of patients with positive JC serology whose JCV could reactivate. This opens up a promising perspective to improve the identification of patients with a higher PML risk, thus, justifying the setting up of larger longitudinal trials among NTZ-treated MS patients.

The cellular production of other cytokines by T-cells, either from the blood or CSF, has been investigated in several studies among patients with NTZ-related PML. Blood T-cell responses were dominated by IL-10 production rather than IFN-γ production, and higher levels of IL-10, IL-5, and IL-15 were detected in the CSF shortly after the diagnosis of PML (64). Starting from the premise that IL-10 production is associated with poor control of viral infections, NTZ-associated PML could be marked by a switch of cytokine production from Th1 to Th2. Given that early false-negative detection of JCV DNA in the CSF by PCR can occur, measuring IL-10, IL-5, and IL-15 in the CSF may help to diagnose PML. Nevertheless, intra cellular cytokine assay seems to be difficult to use in routine analysis.

## Natalizumab and the T-Cell Receptor Repertoire

The T-cell receptor (TCR) repertoire is diverse thanks to the combinatorial rearrangement of TCR genes and their recombination during these rearrangements. Rearrangement of V, D, and J gene segments encodes for CDR3, which is involved in TCR contact with peptide-CMH complex (65). In MS patients, the T-cell repertoire is altered compared to healthy patients (66) with a sharp increase in the frequency of TCR β chain variable region (TCRβV) expansions in the peripheral blood over time (67). NTZ was shown to curb expansion of the TCR repertoire in blood, with a lower proportion of Vbeta elements with TCR repertoire expansions in blood (68). In addition, TCR repertoire alterations in CSF were more pronounced in NTZ-treated than in untreated patients (68). As TCR repertoire diversity plays a role in antiviral immunity (65), NTZ may weaken such antiviral defenses. Interestingly, additional peripheral TCR expansions have been identified 3 months after diagnosis of NTZ-related PML that preceded the development of an immune reconstitution syndrome (IRIS): whether this reflects the recovery of efficient antigen recognition should be investigated further. Overall, though potentially promising, these results should be reproduced. For the moment, however, they are far from being exploitable in routine clinical practice.

## microRNAs and Individual PML Risk Assessment

Epigenetic mechanisms, such as microRNAs (miRNAs) changes, have recently been shown to be involved in MS pathophysiology (69). microRNAs are a class of small, highly conserved, non-coding RNA molecules that control gene expression by binding to complementary sequences in the 3′ untranslated regions of the messenger RNAs (70). They play an important role not only in modulating innate immunity, especially antiviral immunity, but also in adaptive immunity by regulating B- and T-cell development and differentiation (71). The study of B-cells in NTZ-treated patients highlighted that it could modulate the expression of a particular set of deregulated miRNAs found in untreated MS patients (72). Within the first 6 months of treatment, a decrease in let-7c, miR-125a-5p, and an increase in miR-642 expression have been found (73). Interestingly, miR-125a-5p could play a role in the leukocyte migration process and in the regulation of brain endothelial integrity. Furthermore, it has been suggested that three other miRNAs, miR-320, miR-320b, and miR-629, are related to PML: miR-320 and miR-320b showed higher expression and miR-629 lower expression in PML patients than in non-PML patients (73). Remarkably, it has been suggested that miR-320b may have “virus–host interaction,” thus, its higher expression in anti-JCV antibody-positive patients pave the way for its putative relationship with the virus and PML (73). Although miRNAs have been linked to B virus chronic infection, the possible role of miRNAs in viral infection remains to be confirmed. Finally, miR-320 and miR-320b have binding sites in L-selectin, another possible biomarker of PML. Whether this indicates a direct link between the two markers, miR-320 and miR-320b impacting the interaction between L-selectin and the endothelial cells, remains to be assessed. Another micro RNA, miRNA-126, has been associated with PML (74), and could point to a relevant pathophysiological interaction with PML onset by acting on JCV replication: Mir-126 blockade was found to increase POU2AF1 (75) a crucial regulator of the transcription factor Spi-B (76), which is involved in early JCV gene expression and JCV activity (77).

Overall, whether these miRNAs will finally serve as biomarkers for the early detection of PML in NTZ-treated patients in the future remains to be assessed in a longitudinal clinical setting (Figure 1).

## Perspectives and Conclusion

Despite potential life-threatening side effects such as PML, NTZ is one of the most effective therapies for RR-MS. Estimating an individual’s risk of PML is still a major challenge, and therapeutic strategies are mainly guided by an overall estimation of PML probability. Up to now, monitoring is currently based on clinical data (NTZ exposure and prior use of immunosuppressive drugs), MRI scanning and presence of JCV-specific antibodies. Adding the anti-JCV antibody index in patients without prior use of immunosuppressive drugs to better determine PML risk is now a promising alternative. Although this marker needs to be further validated, a high anti-JCV antibodies index (>1.5) encourages extreme caution, and if treatment is maintained, justifies a narrow clinical and MRI follow-up. In case of PML suspicion, NTZ should be immediately stopped and CSF analyzed for JCV DNA screening and eventually determination of CSF JCV antibody index. Interestingly, several novel immunological biomarkers that could help to identify patients with a high risk of PML more accurately have been identified. These include selectin-positive circulating T-lymphocytes, JCV effector memory T-cells, or miRNA levels (Figure 1). The real predictive value of these markers still needs to be investigated in clinical trials. Though it is not yet possible to accurately predict the risk of PML in individual patients, these new markers have paved the way for «new combined monitoring», which will include immunological screening, in NTZ-treated patients.

## Conflict of Interest Statement

The authors declare that the research was conducted in the absence of any commercial or financial relationships that could be construed as a potential conflict of interest.
